# An evaluation of designs for Phase I/IIa dose-finding studies in Tuberculosis

**DOI:** 10.1177/09622802261430905

**Published:** 2026-06-01

**Authors:** Alessandra Serra, Pavel Mozgunov, Patrick PJ Phillips, Thomas Jaki

**Affiliations:** 1 47959 University of Cambridge, MRC Biostatistics Unit, UK; 2 UCSF Center for Tuberculosis, 166668 University of California, USA; 3 Faculty of Informatics and Data Science, University of Regensburg, Germany

**Keywords:** Adaptive designs, infectious diseases, dose-finding, dual endpoint, early activity

## Abstract

Early development of tuberculosis (TB) treatments often separates dose-finding in healthy volunteers (Phase I) and safety and early activity in patients (Phase IIa). The BTZ-043 study (NCT04044001) is a recent study that combined these two objectives in patients in a single study. In this work, we describe and compare three different design options which consider safety and activity endpoints differently in the dose escalation process. In simulations we show that the design that incorporates information about activity together with safety in the dose escalation process allows more precise estimation of the optimal dose and leads to a higher power on average in selecting at least one suitable dose at the end of the study compared to the design that considers only the safety endpoint for dose escalation.

## 1. Introduction

Tuberculosis (TB) remains the world’s second leading cause of death from a single infectious agent.^1^ The development of novel TB drugs has recently progressed and a new consortium for clinical drug development in TB was founded in 2021.^2^ The UNITE4TB Consortium aims to address major challenges in the design and development of clinical trial designs from innovative Phase II designs to large scale Phase III trials.

One of the challenges relates to the design of Phase IIa trials, which aim to investigate the early bactericidal activity (EBA)^3^ of an agent. These are the first clinical studies that evaluate whether the drug is active in a small group of patients which are randomised to different doses of the drug. These studies are preceded by healthy volunteer Phase I dose-escalation trials that aim to identify the maximum tolerated dose (MTD) of a drug, which is the dose with a dose-limiting toxicity probability closest to a target toxicity value. Generally, standard early phase trials in TB setting consist of dose-ranging studies where all patients (approximately 10–15 patients per group) receive a specific dose of the experimental or control compounds. Descriptive studies with no inferential statistics or hypothesis testing are normally conducted.^3,4^ However, several studies have also reported results of statistical tests used to compare activity levels between arms. More details can be found in a recent systematic review that has been conducted to compare the methodological aspects of these trials.^5^

In other clinical trial settings (e.g. oncology trials), early phase trials are performed directly in small groups of patients, due to the high toxicities of the drugs,^6^ and they can be broadly divided into three classes of designs: the rule-based, the model-based and the model-assisted designs.^7^ The first ones assign the participants to doses following pre-specified rules that are based on actual observation of target events in the observed data, without assuming any parametric dose-toxicity model. The model-based designs^8^ instead aim to model a dose–toxicity relationship and to update it after observing the data in order to determine the dose for the next cohort of patients. The model-assisted^7,9^ designs do not impose a particular shape on the dose-toxicity and the decision rules can be pre-tabulated and derived from a statistical model. These model-assisted and model-based designs can improve efficiency and accuracy in finding the maximum tolerated dose compared to the rule-base designs, but sometimes they are not used, as they might be less straightforward to implement compared to the conventional rule-based designs. Early phase oncology trials can be further improved by making efficient use of the collected data in order to combine information coming from the safety assessment and the early activity component of the drug in making dose-escalation and de-escalation decisions. The so-called seamless phase I/II trials, which combine safety and activity assessments in one single protocol, might provide potential to improve dose-finding, despite drug activity may require longer observation windows, meaning that phase I/II designs might prolong trial duration (given the same sample size).^10^

In TB, a small number of clinical trials have adopted these novel adaptive designs^11^ and only a model-based adaptive design has been implemented for dose finding (ClinicalTrials.gov ID: NCT04044001^12^) in participants with newly diagnosed pulmonary TB. The aim of this paper is to compare different dose escalation trial designs that use either safety alone or safety and EBA assessment within a single trial to increase the efficiency by trying to define the optimal dose rather than just the maximum tolerated dose. In particular, we will consider the designs which are summarised in Table 1. Our comparison of designs is informed by the recently completed TB trial (NCT04044001, described in Section 2) which evaluated safety and early activity of a novel compound in TB patients. Thus, dose escalation designs that consider both endpoints in a model-based dose escalation framework^13^ are explored in this work as no previous work has explored these types of designs in the specific context of a Tuberculosis setting.

**Table 1.** Description of the considered dose-escalation designs.

Design	Phases and endpoints	Selection of doses for Phase II
BLRM–NCT04044001 study	Phase I: dose escalation based on safety; Phase II: evaluates EBA	3 pre-specified doses
BDEM	Phase I: dose escalation based on safety and EBA; Phase II: evaluates EBA	3 pre-specified doses
BDEM_T	Phase I: dose escalation based on safety and EBA; Phase II: evaluates EBA	3 doses closer to MED
BDEM_O	Seamless Phase I/II: dose escalation based on safety and EBA	–

EBA: early bactericidal activity; MED: minimum effective dose.

The rest of the manuscript continues as follows: in Section 2 the motivating clinical trial is introduced before a detailed explanation of the different designs is provided in Section 3. In Section 4, a numerical evaluation in the setting of the motivating example is provided. We conclude with a discussion in Section 5.

## 2. Motivating trial

The BTZ-043 study (ClinicalTrials.gov ID: NCT04044001^12^) is a Phase I/IIa trial that evaluates the safey and early activity of the BTZ-043 compound in persons with TB. The study is divided into two parts.

The objective of the first part (Phase I) was to assess the safety and tolerability of BTZ-043 and was designed using a two-parameter Bayesian logistic regression model-based design (BLRM).^14^ Participants were treated for 14 days and screened undergoing safety assessments, sputum sampling and additional pharmacokinetic evaluations. Patients could be assigned to several doses (250, 500, 750, 1000, 1250, 1500, 1750, 2000 mg) of the drug in cohorts of 3. The choice of dose was based on the data of previous patients and a parametric dose-toxicity model (using the MoDEsT web app^15^) was used to make a recommendation to the Trial Steering Committee (TSC) regarding whether to escalate, de-escalate or continue with the same dose for the next cohort of patients. Safety data for a current cohort of patients was reviewed after all patients in the cohort have completed at least 7 days of dosing. The starting dose was 250 mg and the aim of the adaptive dose-escalation algorithm was to identify the dose that was closest to the safety toxicity target of 10%. Thus, the primary endpoint for this phase was a binary endpoint (yes/no) to indicate whether a dose-limiting toxicity (DLT) within 7 days was observed. Up to 33 patients were considered in this part of the study. At the end of the study, all doses up to 1750 mg were found to be safe. The highest dose (2000 mg) showed a plateau in the achieved exposures and no obvious increase in bacterial killing and thus no further escalation was recommended.

In the second part (Phase IIa) of the trial, the aim was to explore the early activity of the drug. Patients were randomised to receive one of the three pre-specified doses and the standard of care arm with a ratio of 3:3:3:2 and a total of 53 patients. When the trial was started the protocol was mentioning a ‘low’, ‘medium’, and ‘high’ dose of BTZ-043 for this stage. As the trial progressed, a protocol amendment was put through based on early data from Phase I (mainly pharmacokinetics data that was showing the exposure plateau) to target 250, 500 and 1000 mg doses, as it was clear that exposure did not increase beyond 1000 mg. Overnight sputa were collected on several days during the days of the exposure of the treatment. Time to positivity (TTP) in mycobacterial liquid culture was measured for each collected sputum sample.^16^ The EBA of different doses was measured by estimating the average slope of change in time to positivity between sputa collected in day 1 and in day 14. Each patient had a 7 days post-treatment follow-up visit.

In the BTZ-043 study, the choice of the doses to continue to the second stage was based on safety and some pharmacokinetics considerations, but the EBA data was not formally incorporated inside the dose escalation process. To be able to make a more accurate choice of the doses and thus potentially improve the efficiency of the overall trial, we will explore different dose-escalation designs that combine safety and EBA information inside the dose escalation process in order to identify an optimal dose – based not only on safety but also on EBA – at the end of the trial. Toxicity and activity will be considered to be non-decreasing functions with increasing doses and several metrics will be compared among those designs.

## 3. Methods

In this section, we provide the details of three different design options that will be compared in the simulation study presented in Section 4.

### 3.1. BTZ-043 original study design – BLRM

Consider the setting of the trial described in Section 2, where K are the actual active doses, $d_{k},k\in \{1,\ldots ,K\}$ . The control dose is denoted by $d_{0}$ , but this is not considered in the escalation process. Assume that a patient’s outcome, a safety endpoint, follows a binomial distribution, thus $T^{\left(k\right)}∼Bin(p(d_{k}))$ . Let denote by $n_{k}$ the number of patients that have been assigned to dose $d_{k}$ and $t_{k}$ the number of dose-limiting toxicities observed at dose $d_{k}$ . Patients are recruited in cohorts and let denote by $D_{N}=\{(n_{k},t_{k}),k=1,\ldots ,K\}$ the data accumulated up to cohort N with a total number of $N_{1}$ patients recruited. Let $D_{N_{1}}$ be the total data accumulated at the end of Phase I.

A two-parameter logistic regression (BLRM)^14^ is used to model the dose–toxicity relationship. The model is defined as

$$p(d_{k})=\frac{\exp\;\{\phi_{1}+\phi_{2}\log\;\left(d_{k}\right)\}}{1+\exp\;\{\phi_{1}+\phi_{2}\log\;\left(d_{k}\right)\}}$$

where $\phi_{1}$ and $\phi_{2}$ are the parameters of the model. A Bayesian approach to inference is considered in this work. This consists on a probability density function, that is $f_{T^{\left(k\right)}}(d_{k},\theta )$ , where $\theta =(\phi_{1},\phi_{2})$ and a prior distribution π(θ) . Then, the posterior distribution $\pi (\theta |D_{N})$ is obtained after observing the accumulated data ( $D_{N}$ ) up to cohort N .

The prior distribution for the parameters in the model, $\phi_{1}$ and $\phi_{2}$ , is expressed in terms of pseudo data that represents hypothetical data, collected in relevant clinical trials or coming from experts’ opinion, observed before the start of the trial. This translates into assuming to have collected $n_{K}$ patients on the highest dose $d_{K}$ and $n_{1}$ patients on the lowest dose $d_{1}$ and having observed $t_{1}$ and $t_{K}$ toxicities on these doses. Then assuming that

$$\left\{ \begin{array}{l} p(d_{1})∼Beta(t_{1},n_{1}-t_{1})\\ p(d_{K})∼Beta(t_{K},n_{K}-t_{K}) \end{array}\right.\;$$

and that the two beta distributions are independent, a joint prior distribution of π(θ) can be found and details are provided in Whitehead and Willamson.^17^

The next dose $d_{k^{⋆}}$ that is allocated to the next cohort of patients N+1 in the dose escalation process, is the one whose estimated risk of toxicity is closest and less than a target toxicity level γ . Thus,

$$d_{k^{⋆}}={\arg\;\max}_{k^{⋆}\in \{1,\ldots ,K\}}\;\{\hat{p}(d_{k}|D_{N})\leq \gamma \}$$

where $\hat{p}(d_{k}|D_{N})=E[p(d_{k}|D_{N})]$ . The escalation process terminates when a minimum overall number of patients $N_{1}$ have been enrolled. Additional stopping rules can also be considered (e.g. stopping the escalation when more than a pre-specified number of patients have been allocated to a dose or safety rules in order to limit the exposure of patients to high toxic doses^18,19^), but these were not implemented in this work. The aim of this phase is to find at the end of the study the dose that is closer to the target toxicity value, which is the maximum tolerated dose (MTD).

At the end of the first phase of the trial, three pre-specified doses (e.g. ${\tilde{d}}_{1}<{\tilde{d}}_{2}<{\tilde{d}}_{3}$ , where ${\tilde{d}}_{k}\in \{d_{k},k\in \{1,\ldots ,K\}\}$ ) are considered to continue to the next stage. These doses are fixed after evaluating early data from the first phase of the trial. If at the end of phase I they are indeed found to be safe, then all of them are tested. If instead, safety is a concern for some of them, then only doses below the MTD are considered to continue to the next phase.

Assume that a patient’s secondary outcome, an activity endpoint, follows a normal distribution, thus $Y^{\left(k\right)}∼N(\mu^{\left(k\right)},\sigma^{2}),k\in \{0,1,2,3\}$ , with known σ . Patients are allocated to these doses and the control dose $d_{0}$ with a ratio of 3:3:3:2 in favour of the active doses and a total of $N_{2}$ patients. No interim analyses are planned for this phase. Let denote by $D_{N_{1}+N_{2}}$ the total data accumulated at the end of Phase II.

In the original study design, there was no formal statistical test to compare each active dose to the control dose. However, in this work, in order to estimate the minimum effective dose (MED), that is the lowest dose that shows an improvement in terms of activity compared to the control one, we consider a hierarchical step-down Dunnett test.^20^ The rejection procedure is presented in Section 3.5.

### 3.2. Design with dual-endpoint dose escalation – BDEM

The first design to be compared to the BTZ-043 original study consists of two parts. In the first part, differently from the original study design, the dose escalation is performed considering a dual-endpoint, which combines information about the safety of the drug together with the early activity information at a given dose. The evaluation of both safety and EBA in the dose-escalation process might result in a more accurate choice of the optimal dose with the best safety/activity trade-off. However, in terms of trial duration, this design might require longer time (up to 7 days more per participant as 7 days of treatment are required for the safety evaluation and 14 days of collection of the sputum and then 6–8 weeks for cultures to grow for the activity endpoint) in order to evaluate both safety and EBA endpoints for each cohort of patients. The second part of the study is the same as per the original BTZ-043 study, which is described in Section 3.1.

In order to incorporate the information of early activity in the dose escalation process, a dual-endpoint is considered here. Only the toxicity endpoint is modelled using a Bayesian logistic regression model (BLRM) as described in Section 3.1. This consists on using a two-parameter logistic regression model to describe the dose–toxicity relationship. The activity endpoint is modelled using a flexible Gaussian random walk. Thus, the full model comprises a BLRM for toxicity and a continuous response model for activity and we refer to this as ‘Bayesian dual-endpoint model’ (BDEM).

To describe the dose–activity relationship, a first order random-walk structure is used^21,22^ to model the mean activity outcomes. As shown in Yeung et al.,^22^ this model allows to have a flexible non-parametric activity model so that non-monotone dose–activity relationship curves can be captured. Thus,

$$\mu^{\left(k\right)}-\mu^{\left(k-1\right)}∼N(0,(d_{k}-d_{k-1})\sigma_{\mu }^{2})$$

The toxicity and activity endpoints can be then modelled separately or jointly. There are other works that consider dual endpoint modelling in dose escalation – for example Bekele and Shen (2005)^21^ characterised toxicity and activity by a joint framework with a correlation structure.

The rule used in this design to choose the next dose $d_{k^{⋆}}$ for the next cohort of patients N+1 is based on the posterior estimate of the activity mean value being between two target values $\epsilon_{1},\epsilon_{2}$ , which refer to the minimum and maximum secondary endpoint values that are of interest to target for the study (the choice of these parameters can be elicited from clinicians), and by controlling^14^ that the probability that the risk of toxicity at this dose level exceeds γ is less or equal than $\tilde{\gamma }$ . That translates into choosing $d_{k^{⋆}}$ such that:

$$\epsilon_{1}\leq {\hat{\mu }}_{D_{N}}^{\left(k^{⋆}\right)}\leq \epsilon_{2},\;P(p(d_{k^{⋆}})>\gamma |D_{N})\leq \tilde{\gamma }$$

where ${\hat{\mu }}_{D_{N}}^{\left(k^{⋆}\right)}=E[\mu^{\left(k^{⋆}\right)}|D_{N}]$ . This means that all the doses for which the probability of having an overdose is exceeding a pre-specified level are excluded. Then, among the selected doses, the one with the highest activity response in the target range is selected for the next cohort of patients.

At the end of the first phase of the trial, three pre-specified doses (e.g. ${\tilde{d}}_{1}<{\tilde{d}}_{2}<{\tilde{d}}_{3}$ ) as in the original BTZ-043 study, are considered to continue to the next stage. If at the end of phase I these doses are indeed found to be safe, then all of them are tested. If instead, safety is a concern for some of them, then only doses below the MTD are considered to continue to the next phase. Patients are allocated to the selected doses with $n_{0}$ patients allocated to the control arm and $N_{2}-n_{0}$ patients equally allocated to the selected active doses. The testing strategy used at the end of Phase II is the same as the one described in Section 3.5.

### 3.3. Design with dual-endpoint dose escalation and different choice of doses for Phase II - BDEM_T

The second alternative design consists of two parts. In the first part, differently from the original study design, the dose escalation is performed considering a dual-endpoint, which combines information about the safety of the drug together with the early activity information at a given dose. This part follows the same procedure as described in Section 3.2 and the idea is to efficiently make use of both safety and EBA information in order to find an optimal dose.

At the end of the Phase I part, a different choice of doses, compared to the original study design, are allowed to continue to the Phase II. This modification of the selection of the doses is explored in order to understand whether a more accurate choice of the doses can be made at this point of the trial and thus it leads to a better estimate of the optimal dose at the end of the trial. The three doses that are considered to be safe and for which the posterior estimate of the activity mean value is between the two target values at the end of Phase I are selected. This means finding the doses ${\tilde{d}}_{1}<{\tilde{d}}_{2}<{\tilde{d}}_{3}$ such that:

$$\hat{p}({\tilde{d}}_{k}|D_{N_{1}})\leq \gamma ,\;\epsilon_{1}\leq {\hat{\mu }}_{D_{N_{1}}}^{\left(k\right)}\leq \epsilon_{2},\;k\in \{1,2,3\}$$

Only 3 doses are selected and these are the lowest doses that satisfy the conditions above. In case no dose satisfies the second condition, then all doses that are lower than the maximum tolerated dose are selected. Patients are allocated to the selected doses with $n_{0}$ patients allocated to the control arm and $N_{2}-n_{0}$ patients equally allocated to the selected active doses. The rest of the Phase II is conducted as described in Section 3.5.

### 3.4. Seamless Phase I/II design – BDEM_O

The third design consists of a single study where the dose escalation is conducted for all patients from Phase I and Phase II ( $N_{1}+N_{2}$ patients) using the dual-endpoint model as described in Section 3.2. At the end of the study, the three doses with the highest number of patients allocated to them are compared with the control dose using the testing procedure described in Section 3.5. Patients are allocated to these doses and the control dose $d_{0}$ with a ratio of 3:3:3:2 in favour of the active doses and a total of $N_{2}$ patients. This design is explored in order to see whether it could lead to a more accurate estimate of the optimal dose, as the dose escalation procedure considers both safety and EBA information but in a larger patient population compared to the other designs. However, the dose escalation process for this trial would be longer compared to the other designs (as safety requires at least 7 days of dosing and EBA at least 14 days plus 6–8 weeks).

### 3.5. Hypothesis testing procedures for Phase II

As mentioned above, in the original study design there was no formal statistical test to compare each active dose to the control dose. However, in this work, in order to estimate the minimum effective dose (MED), which is the lowest dose that shows an improvement in terms of activity compared to the control one, we consider a hierarchical step-down Dunnett test.^20^ All data accumulated until the end of the study (that is Phase I and Phase II patient data, with a maximum total of $N_{1}+N_{2}$ patients) is considered for the statistical tests.

Firstly, the highest dose ( $\tilde{d_{3}}$ ) is compared to the control and the following null hypothesis is tested: $H_{03}:\{\mu^{\left(3\right)}-\mu^{\left(0\right)}\leq 0\}$ . If this hypothesis is rejected at level α , than the second hypothesis $H_{02}:\{\mu^{\left(2\right)}-\mu^{\left(0\right)}\leq 0\}$ is tested. If this hypothesis is rejected then the last hypothesis $H_{01}:\{\mu^{\left(1\right)}-\mu^{\left(0\right)}\leq 0\}$ is tested. The procedure stops whenever a hypothesis cannot be rejected and the minimum effective dose is estimated to be the one associated to the last hypothesis that we were able to reject. This rejection procedure will be implemented in the simulation study for all designs and for the design described in Section 3.3 this will be referred as ‘BDEM_T1’.

Additional rejection procedures for the identification of the MED can be implemented in this setting. In this work, we will explore also two other procedures. These additional rejection procedures will be explored in the simulation study only for a specific design as the potential differences and conclusions can also be applied to the other proposed designs. We choose to apply those to the design described in Section 3.3. The so called ‘Dual Lower Test’ (BDEM_TDL) refers to the same rejection procedure as described above, but with an additional constraint that, at the end of the trial, the posterior estimate of the activity mean value of the dose compared to the control arm is above $\epsilon_{1}$ . That is, we firstly consider the null hypothesis $H_{03}:\{\mu^{\left(3\right)}-\mu^{\left(0\right)}\leq 0\}$ . If we can reject the null hypothesis, then we check if

$${\hat{\mu }}_{D_{N_{1}+N_{2}}}^{\left(3\right)}\geq \epsilon_{1}$$

If the above is satisfied, then we proceed similarly for the other hypotheses following the hierarchical testing procedure as described above.

The second is called ‘Dual Lower and Upper Test’(BDEM_TDUL) and it consists on the same rejection procedure as ‘BDEM_T1’ but with an additional constraint that, at the end of the trial, the posterior mean estimate of the activity level of the dose compared to the control arm is above $\epsilon_{1}$ and below $\epsilon_{2}$ . That is, we consider the hypothesis $H_{03}:\{\mu^{\left(3\right)}-\mu^{\left(0\right)}\leq 0\}$ , we check if

$$\epsilon_{1}\leq {\hat{\mu }}_{D_{N_{1}+N_{2}}}^{\left(3\right)}\leq \epsilon_{2}$$

and if the above is satisfied, then we proceed similarly for the other hypotheses following the hierarchical step-down Dunnett testing procedure.

## 4. Numerical evaluation

In this section, we evaluate the operating characteristics of the designs described above under different toxicity and activity scenarios. The numerical results are found using R^23^ and the package *crmPack*^24,25^ which makes use of the package *rjags*^26^ to estimate the posterior distributions using MCMC samples. A thousand of replicate simulations are provided and the code to reproduce the simulations is provided at the link: https://github.com/aspapercode/evalph12design.git

### 4.1. Setting

We consider the following range of doses – 250, 500, 750, 1000, 1250, 1500, 1750 mg – for the escalation process. The highest dose of 2000 mg is not considered as in the BTZ-043 study a further escalation to the maximum dose was not recommended at the end of the process due to a plateau in the achieved exposures and no obvious increase in bacterial killing.

Patients are recruited in cohorts of 3 and up to $N_{1}=33$ patients are considered in this study.

The prior parameters $\phi_{1}$ and $\phi_{2}$ in the toxicity model described in Section 3.1 are expressed in terms of pseudo data (this is informed by the original BTZ-043 study), that is assuming that 3 patients are treated at 250 mg (lowest dose) and 2000 mg (highest dose) with 0.03 and 0.3 dose limiting toxicities (DLTs) observed at these two dose levels. The target toxicity level is set to γ=10% as in the BTZ-043 study.

**Figure 1.**
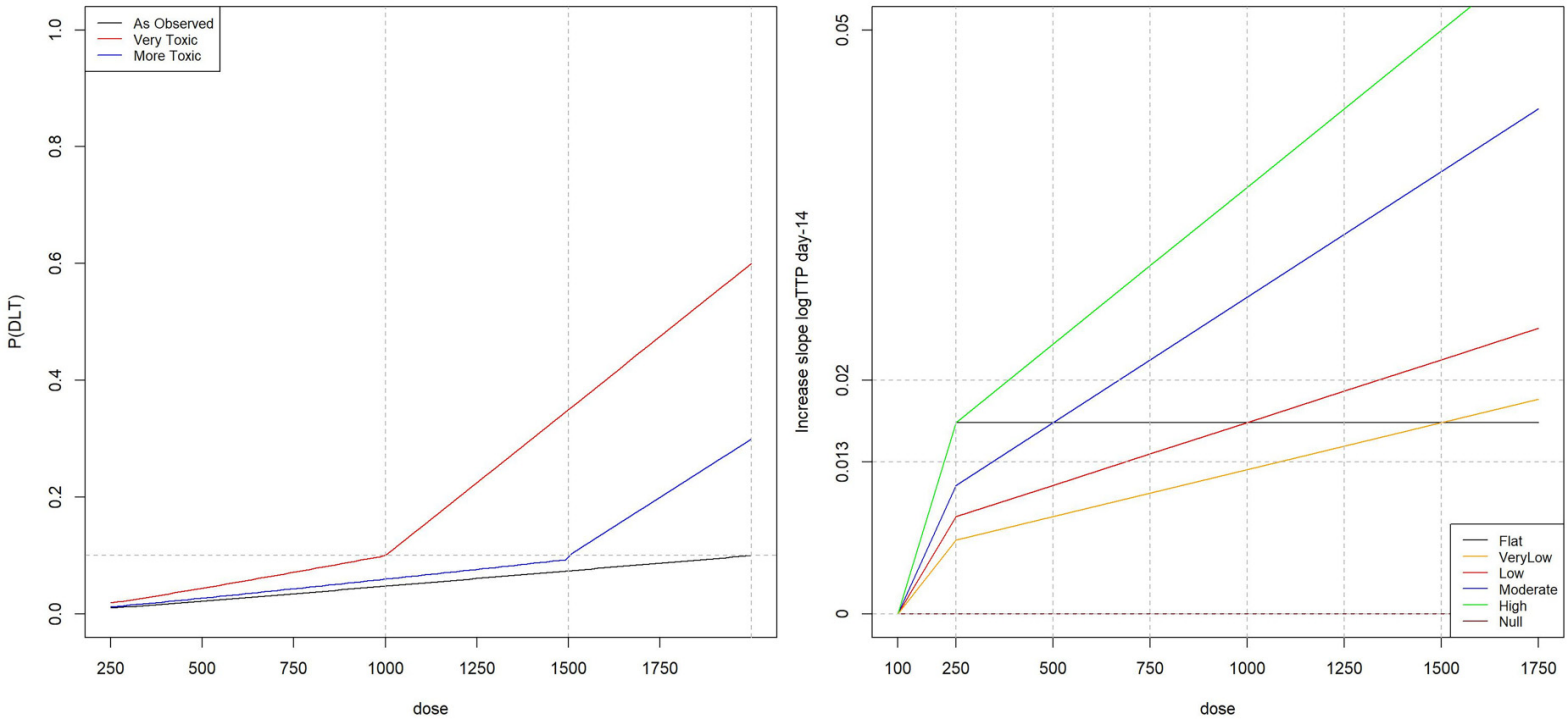
(Left panel) Probabilities of toxicity for the considered range of doses. (Right panel) Different activity scenarios for the considered range of doses.

For the toxicity scenarios, we consider three different cases. Firstly, the same setting as in the original BTZ-043 study where all doses up to 2000 mg were expected to be safe is considered. Secondly, the case where the MTD coincides with 1000 mg is considered and thirdly, the case where 1000 mg is safe but the MTD is 1500 mg. These three different toxicity scenarios for Phase I are summarised on the left panel of Figure 1 and these are referred as:

- ‘As Observed’: this corresponds to the scenario where all doses are considered as safe – the probability of toxicity is below the target value for all doses. Thus, the maximum tolerated dose in this setting is 2000 mg.
- ‘More Toxic’: in this scenario the maximum tolerated dose is 1500 mg.
- ‘Very Toxic’: in this scenario the maximum tolerated dose is 1000 mg.

In regards to the activity scenarios, in order to generate them, we assume that the increase in slope in log(TTP) at day 14 follows a linear relationship with the dose level. The activity–dose relationship is informed by the BTZ-043 study and the linear model is estimated using the summary data available in the Supplementary Material of the BTZ-043 study.^12^ The considered linear model is as follows:

$$\text{Increase slope in }\log\;(\text{TTP}{)}_{14}∼\beta_{0}+\beta_{1}d_{k}$$

The estimated coefficients are ${\hat{\beta }}_{0}=0.0152$ and ${\hat{\beta }}_{1}=1.342857e-06$ . This implies that for the BTZ-043 study no increase in slope in log(TTP) was found among doses. However, the specific values of these parameters can be discussed and perhaps elicited from clinicians. The target activity level is $\tilde{\epsilon }={\hat{\beta }}_{0}+{\hat{\beta }}_{1}{\bar{d}}_{k}=0.0166$ , where ${\bar{d}}_{k}$ is the average value of the active doses. The parameters $\epsilon_{1}$ and $\epsilon_{2}$ are set to be $\epsilon_{1}=\tilde{\epsilon }-0.0035$ and $\epsilon_{2}=\tilde{\epsilon }+0.0035$ in order to create several activity scenarios with different doses with an activity value that is between the minimum and maximum activity values that are of interest to target for this study. For the simulation study, we set the control dose to be equal to 100 mg. This is only used for the final testing procedure in each of the considered designs at the end of Phase II and not in the escalation processes.

Several activity scenarios are considered and summarised on the right panel of Figure 1. These are referred as:

- ‘Flat’: the increase in slope in log(TTP) at day 14 is equal to $\tilde{\epsilon }=0.0166$ for all doses.
- ‘VeryLow’, ‘Low’, ‘Moderate’, ‘High’: the increase in slope in log(TTP) at day 14 is equal to $\tilde{\epsilon }=0.0166$ for doses 1500, 1000, 500 and 250 mg, respectively.
- ‘Null’: the increase in slope is equal to zero for all active doses and the control dose.

For Phase II, the activity data are generated with standard deviation equal to 0.0105 (that is the average standard deviation between standard deviations of outcomes from Phase I and Phase II of BTZ-043 study) and no correlation is assumed between the safety and early activity endpoints. For the dual-endpoint design, we set $\tilde{\gamma }=50\%$ so that there the probability that the next assigned dose is overly-toxic is less than 50%. The pre-specified α level for the testing procedure is set at 5%. Patients are allocated to the selected doses and the control dose $d_{0}$ with a ratio of 3:3:3:2 in favour of the active doses and a total of $N_{2}=53$ patients. In the case where less active doses are taken forward to Phase II, then a total of $N_{2}=53$ patients is still considered with $n_{0}=11$ patients allocated to the control dose and the remaining patients are equally allocated to the selected doses.

**Figure 2.**
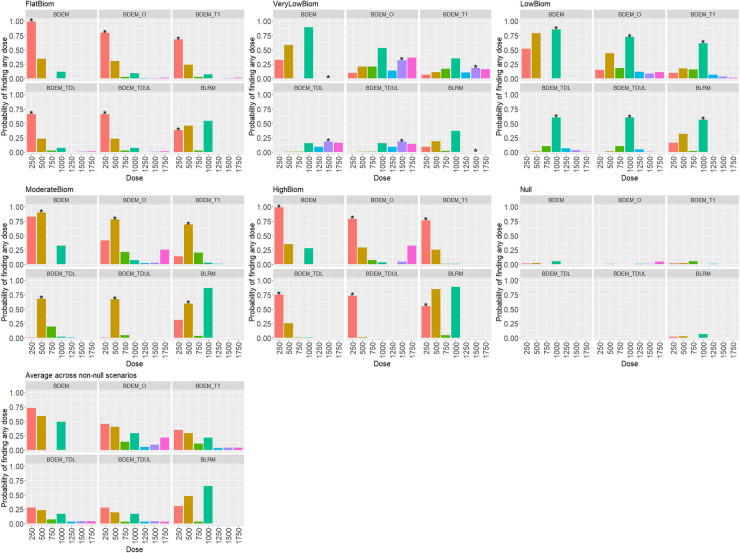
Probability of finding any dose in each activity scenario and each design for the ‘As Observed’ Toxicity scenario. The probabilities of finding the safe dose with activity level equal to $\tilde{\epsilon }$ in each activity scenario are indicated by an asterisk (*). The designs are labelled as described in Section 3.

In the next section, we summarise how the prior parameters are chosen for the dose-activity model.

### 4.2. Calibration of prior for the dose-activity model

The prior parameters used for the dual-endpoint escalation model can be selected from a range of plausible initial guesses. These are the variance of the random walk $\sigma_{\mu }^{2}$ and the variance of the activity $\sigma^{2}$ . A calibration approach^27,28^ has been implemented in order to choose those parameters.

Several values for these parameters were tested, in particular $\sigma^{2}=\sigma_{\mu }^{2}=10^{-6},10^{-5},10^{-4},10^{-3},10^{-2},10^{-1},1,2$ . All the other parameters in the model specifications are set as described in Section 4.1. All activity scenarios, as described in Figure 1, were considered for a given toxicity scenario that is the so called ‘As Observed’, as described on the left panel of Figure 1, which reflects the toxicity scenario used in the original BTZ-043 study. At the end of the calibration approach, the final parameters are chosen in order to maximise the geometric mean of probabilities of correct selections across scenarios at the end of Phase II using the design described in Section 3.3. For each activity scenario described in Figure 1, the probability of correct selection is the proportion of rejections of the hypothesis that compares the control arm and the dose for which the increase in slope in log(TTP) at day 14 is equal to $\tilde{\epsilon }$ .

The results of the calibration procedure are summarised in Table A.3. The maximum value for the geometric mean of proportions of correct selections is found to be 0.47 and thus, the following parameters were chosen in this work: $\sigma^{2}=10^{-5}$ and $\sigma_{\mu }^{2}=10^{-3}$ .

### 4.3. Metrics and operating characteristics

The following metrics are compared among the different designs:

- the estimated maximum tolerated dose (MTD) at the end of Phase I or end of the study for the seamless Phase I/II design;
- the estimated optimal dose – that is the minimum dose that satisfies the safety and activity constraints, thus the minimum dose k∈{1,…,K} such that $p(d_{k}|D_{N_{1}})\leq \gamma$ and $\epsilon_{1}\leq {\hat{\mu }}_{D_{N_{1}}}^{\left(k\right)}\leq \epsilon_{2}$ – at the end of Phase I or end of the study for the seamless Phase I/II design;
- the estimated minimum effective dose (MED) at the end of Phase II or end of the study;
- the probability of finding at least one dose, all doses (with the condition that the MED is above the lower activity level $\epsilon_{1}$ ) and probability of finding the safe dose with activity level equal to $\tilde{\epsilon }$ (considered to be the correct and the optimal dose that is the target in this study);
- the average power, expressed as the average probability across all non-null activity scenarios of finding at least one dose, all doses or any dose at the end of Phase II.

### 4.4. Numerical results

**Figure A.1.**
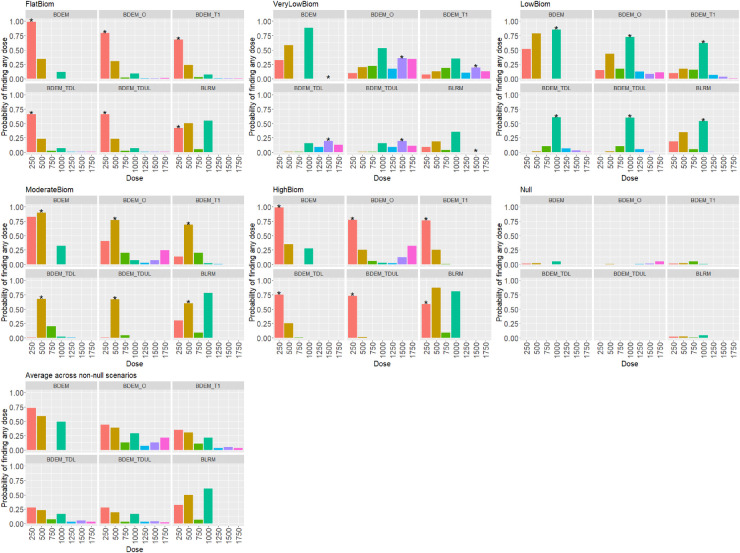
Probability of finding any dose in each activity scenario and each design for the ‘More’ Toxic scenario. The probabilities corresponding to the correct dose in each activity scenario are indicated by an asterisk (*).

In this section, we provide the results of the simulations for each activity scenario and for the toxicity scenario where all doses are safe to reflect the same setting as in the original BTZ-043 study. Similar results and patterns are observed under the other two toxicity scenarios (‘More’ and ‘Very’ toxic) – see results in Tables A.1, A.2 and Figures A.1, A.2 in the Appendix.

Figure 2 reports the probability of finding any dose at the end of Phase II for each activity scenario and each method given the toxicity scenario where all doses are considered safe. It also provides the average power across scenarios for each design. Table 2 provides a detailed summary of all other operating characteristics of the approaches as described in Section 4.2.

**Table 2.** Probability of finding at least one dose (AtLeastOne), probability of finding all doses (All), probability of finding the dose with biomaker level at $\tilde{\epsilon }$ (TargetBmk), minimum effective dose (MED), minimum tolerated dose (MTD), optimal dose at the end of Phase I in terms of activity and safety (OptimalDose) and average power across non-null activity scenarios for each design and activity scenarios under the ‘As Observed’ toxicity scenario. In the first design also the true MTD and MED are reported for each activity scenario.

BLRM – ‘As Observed’ Toxicity						
	AtLeastOne	All	TargetBmk	MED	MTD	True MED (MTD)
FlatBiom	0.60	0.38	0.38	448	1449	250 (1000)
VeryLowBiom	0.00	0.00	0.00	697	1440	1250 (1000)
LowBiom	0.58	0.00	0.57	667	1457	750 (1000)
ModerateBiom	0.93	0.00	0.60	592	1462	500 (1000)
HighBiom	0.98	0.55	0.55	425	1465	250 (1000)
Null	0.07	0.02	0.02	746	1472	
Average Power	0.62	0.19	0.42			
BDEM – ‘As Observed’ Toxicity						
	AtLeastOne	All	TargetBmk	MED	MTD	OptimalDose
FlatBiom	1.00	0.99	0.99	253.76	409	393.25
VeryLowBiom	0.00	0.00	0.00	591	1331	1278.00
LowBiom	0.86	0.00	0.86	462	1069	957.50
ModerateBiom	0.91	0.00	0.90	294	828	551.50
HighBiom	1.00	0.99	0.99	252	669	319.50
Null	0.05	0.01	0.01	784	1749	1748.50
Average Power	0.75	0.40	0.75			
BDEM_T – ‘As Observed’ Toxicity and One Test						
	AtLeastOne	All	TargetBmk	MED	MTD	OptimalDose
FlatBiom	1.00	1.00	0.68	383	409	393.25
VeryLowBiom	0.44	0.44	0.18	1137	1331	1278.00
LowBiom	0.86	0.80	0.62	885	1069	957.50
ModerateBiom	0.90	0.85	0.69	525	828	551.50
HighBiom	1.00	1.00	0.77	311	668	319.50
Null	0.05	0.01	0.01	662	1749	1748.50
Average Power	0.84	0.82	0.59			
BDEM_T – ‘As Observed’ Toxicity and Double Lower Test						
	AtLeastOne	All	TargetBmk	MED	MTD	OptimalDose
FlatBiom	0.98	0.98	0.66	386	409	393.25
VeryLowBiom	0.43	0.43	0.18	1385	1331	1278.00
LowBiom	0.80	0.78	0.60	1007	1069	957.50
ModerateBiom	0.89	0.88	0.68	565	828	551.50
HighBiom	0.99	0.99	0.75	312	668	319.50
Null	0.00	0.00	0.00	NA	1749	1748.50
Average Power	0.82	0.81	0.57			
BDEM_T – ‘As Observed’ Toxicity and Double Lower and Upper Test						
	AtLeastOne	All	TargetBmk	MED	MTD	OptimalDose
FlatBiom	0.98	0.98	0.66	386	409	393.25
VeryLowBiom	0.40	0.40	0.18	1370	1331	1278.00
LowBiom	0.74	0.73	0.60	977	1069	957.50
ModerateBiom	0.71	0.70	0.67	512	828	551.50
HighBiom	0.74	0.74	0.73	254	668	319.50
Null	0.00	0.00	0.00	NA	1749	1748.50
Average Power	0.72	0.71	0.57			


BDEM_O – ‘As Observed’ Toxicity						
	AtLeastOne	All	TargetBmk	MED	MTD	OptimalDose
FlatBiom	1.00	0.80	0.79	327	414	402.25
VeryLowBiom	0.68	0.00	0.32	953	1478	1376.50
LowBiom	0.96	0.04	0.72	720	1151	1005.25
ModerateBiom	0.98	0.07	0.78	466	891	549.50
HighBiom	1.00	0.86	0.79	325	757	311.00
Null	0.05	0.00	0.00	1567	1750	1750.00
Average Power	0.92	0.35	0.68			

It can be observed that the BLRM design, on average and for each activity scenario, provides a 38% to 60% chance of finding the correct dose at the end of Phase II, except for the ‘VeryLow’ activity scenario, where per design, there is no chance to correctly find the dose of 1500 mg. In contrast, for the BDEM design, the chance of finding the correct dose at the end of Phase II varies from 86% to 99% for each activity scenario, except for the ‘VeryLow’ activity scenario where, as before, there is no chance to correctly find the dose of 1500 mg. For the BDEM_T1 design, on average the chance of finding the correct dose at the end of Phase II varies from 18% to 77% for each activity scenario. Here, the probability of finding the dose 1500 mg in the ‘VeryLow’ activity scenario is 18%. Finally, for the seamless design (BDEM_O) the probability of finding the correct dose ranges from around 32% to 79%.

On average across all activity scenarios, the design that incorporates the information about activity in the dose escalation process (BDEM) provides a gain of around 13%, 21% and 33% in the probability of finding at least one dose, all doses and the correct dose, respectively compared to the design that only uses safety data in the escalation process (BLRM). The design that incorporates the information about activity in the dose escalation process, but allows three doses that are closer to the activity level to go to Phase II (BDEM_T1), provides a gain of around 9%, 42% in the probability of finding at least one dose, all doses, respectively compared to the BDEM design. However, the probability of finding the correct dose is decreased by 16% compared to the BDEM design. The design that considers more constraints in the rejection procedure (BDEM_T with Double Lower or Lower and Upper test) has similar operating characteristics as for the BDEM_T with no constraints, but on average slightly lower power across the activity scenarios. The seamless design is the one that leads to the highest probability of finding at least one dose - 30% increase compared to the BLRM design.

In terms of estimation of the MTD, the BLRM underestimates it for all activity scenarios (the MTD is estimated to be around 1500 mg instead of 2000 mg). All the other designs that consider a dual-endpoint in the escalation process provide the same estimates of the MTD for each activity scenario. This is always estimated to be below the true MTD of 2000 mg and the estimate is lower than the estimated MTD in the BLRM design as additional constraints in the escalation process (EBA information is included for the choice of the doses that are selected for the next cohort of patients) need here to be taken into account. The estimation of the optimal dose, that is the minimum dose that is estimated to be safe and active at the end of Phase I, is quite accurate for each dual-endpoint escalation procedure. In terms of estimate of the minimum effective dose (MED), the BLRM and BDEM provide a less accurate estimation compared to the BDEM_T design. The seamless design is the one that provides the most accurate estimate of the optimal dose in each activity scenario despite it underestimates the MED.

Overall, the designs that incorporate information about the safety and the early activity of a compound provide higher chances (up to 33%) of finding the correct dose at the end of the escalation process and they do provide an estimate of the optimal dose (that is the one that satisfies safety and activity constraints).

## 5. Discussion

The aim of this work was to compare different dose escalation designs in the setting of a current TB trial in order to potentially increase the efficiency of the overall study by trying to define the optimal dose rather than just the maximum tolerated dose.

It has been shown that the designs that incorporate the information about the EBA outcome in the dose escalation process allow us to obtain an accurate estimate of the optimal dose and on average higher chances to select at least one suitable dose at the end of the study compared to the design that considers only the safety endpoint for the dose escalation. Overall, it has been found that the seamless Phase I/II trial is the one that leads to the most accurate estimation of the optimal dose despite it underestimates the minimum effective dose. The design that incorporates information about the EBA in the dose escalation process and allows the selection of the three doses that are closer to the activity target level (BDEM_T) shows similar probabilities to select the correct doses to the seamless design.

In this work, we have investigated and compared different designs in the setting of a specific TB study. However, further evaluations might be necessary in other TB trial settings. The exploration of these novel methodologies in this disease area is encouraged, as these novel designs might support better decision-making on optimal doses to be tested in later phases of a novel regimen development. These approaches, which combine safety and early activity information, can be efficient in the drug development process as they allow to find an optimal dose and the exploration of doses, which might be safe but provide less activity, can be reduced. They do make efficient use of all available data for decision-making and they allow to gain more knowledge and information on the range of doses that are closer to the target. On the other hand, however, these designs might result on a significant amount of additional time that is required in order to observe both endpoints, as incorporating activity into the decision process adds at least 6 to 8 weeks before each decision (e.g. for the specific trial setting explored here, these dual-endpoint approaches might impact the duration of the whole trial in roughly additional 2 years compared to the original study). Thus, trial specific considerations might be necessary in order to fully evaluate potential benefits these designs can provide.

## Figures and Tables

**Figure A.2.**
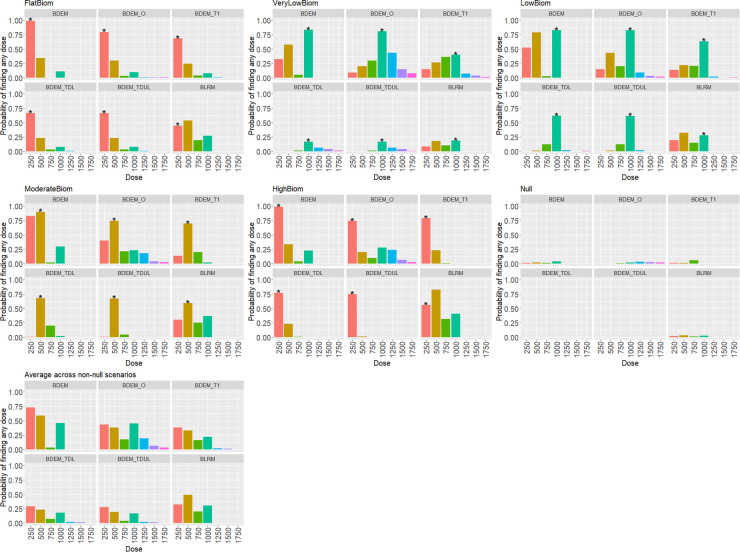
Probability of finding any dose in each activity scenario and each design for the ‘Very’ Toxic scenario. The probabilities corresponding to the correct dose in each activity scenario are indicated by an asterisk (*).

## Abbreviations

EBA: early bactericidal activity
BLRM: Bayesian logistic regression model

## Appendix

**Table A.1.** Probability of finding at least one dose (AtLeastOne), probability of finding all doses (All), probability of finding the dose with biomaker level at $\tilde{\epsilon }$ (TargetBmk), minimum effective dose (MED), Minimum Tolerated Dose (MTD), optimal dose at the end of Phase I in terms of activity and safety (OptimalDose) and average power across non-null activity scenarios for each design and activity scenarios under the ‘More’ toxic scenario.

	AtLeastOne	All	TargetBmk	MED	MTD	OptimalDose
BLRM – ‘More’ Toxic						
FlatBiom	0.65	0.42	0.42	447.31	1223.84	
VeryLowBiom	0.00	0.00	0.00	702.82	1226.54	
LowBiom	0.59	0.00	0.54	635.96	1237.98	
ModerateBiom	0.92	0.00	0.60	580.98	1220.60	
HighBiom	0.98	0.59	0.59	402.55	1221.27	
Null	0.05	0.02	0.02	680.56	1228.14	
Average Power	0.63	0.20	0.43			
BDEM – ‘More’ Toxic						
FlatBiom	1.00	0.99	0.99	253.76	407.75	393.00
VeryLowBiom	0.00	0.00	0.00	589.88	1304.00	1248.50
LowBiom	0.86	0.00	0.86	461.73	1058.50	956.75
ModerateBiom	0.91	0.00	0.90	293.81	810.00	554.00
HighBiom	1.00	0.99	0.99	252.01	650.50	320.50
Null	0.05	0.01	0.01	783.65	1699.75	1698.50
Average Power	0.75	0.40	0.75			
BDEM_T – ‘More’ Toxic and One Test						
FlatBiom	0.99	0.99	0.68	382.77	407.75	393.00
VeryLowBiom	0.30	0.30	0.19	1102.56	1304.00	1248.50
LowBiom	0.85	0.79	0.62	884.65	1058.50	956.75
ModerateBiom	0.90	0.85	0.69	524.75	810.00	554.00
HighBiom	1.00	1.00	0.77	311.18	650.50	320.50
Null	0.06	0.01	0.01	654.55	1699.75	1698.50
Average Power	0.81	0.78	0.59			
BDEM_T – ‘More’ Toxic and Double Lower Test						
FlatBiom	0.97	0.97	0.66	385.46	407.75	393.00
VeryLowBiom	0.28	0.28	0.19	1360.72	1304.00	1248.50
LowBiom	0.79	0.77	0.61	1005.88	1058.50	956.75
ModerateBiom	0.89	0.88	0.68	564.60	810.00	554.00
HighBiom	0.99	0.99	0.75	312.12	650.50	320.50
Null	0.00	0.00	0.00	NA	1699.75	1698.50
Average Power	0.79	0.78	0.58			
BDEM_T – ‘More’ Toxic and Double Lower and Upper Test						
FlatBiom	0.97	0.97	0.66	385.46	407.75	393.00
VeryLowBiom	0.28	0.28	0.19	1349.09	1304.00	1248.50
LowBiom	0.75	0.73	0.60	977.24	1058.50	956.75
ModerateBiom	0.71	0.70	0.67	512.25	810.00	554.00
HighBiom	0.74	0.74	0.73	254.37	650.50	320.50
Null	0.00	0.00	0.00	NA	1699.75	1698.50
Average Power	0.69	0.69	0.57			
BDEM_O – ‘More’ Toxic						
FlatBiom	0.99	0.80	0.79	328.49	413.50	402.50
VeryLowBiom	0.50	0.01	0.35	933.43	1459.50	1348.25
LowBiom	0.95	0.04	0.72	721.88	1142.25	998.75
ModerateBiom	0.97	0.09	0.77	469.79	878.50	550.50
HighBiom	0.99	0.88	0.78	352.96	741.25	310.00
Null	0.02	0.00	0.00	1583.33	1713.75	1713.75
Average Power	0.88	0.36	0.68			

**Table A.2.** Probability of finding at least one dose (AtLeastOne), probability of finding all doses (All), probability of finding the dose with biomaker level at $\tilde{\epsilon }$ (TargetBmk), Minimum Effective Dose (MED), Minimum Tolerated Dose (MTD), optimal dose at the end of Phase I in terms of activity and safety (OptimalDose) and average power across non-null activity scenarios for each design and activity scenarios under the ‘Very’ toxic scenario.

	AtLeastOne	All	TargetBmk	MED	MTD	OptimalDose
BLRM – ‘Very’ Toxic						
FlatBiom	0.66	0.45	0.45	395.23	804.12	
VeryLowBiom	0.00	0.00	0.00	648.88	810.29	
LowBiom	0.43	0.00	0.28	568.90	803.85	
ModerateBiom	0.80	0.00	0.59	509.60	801.24	
HighBiom	0.93	0.56	0.56	390.71	815.67	
Null	0.05	0.02	0.02	591.84	797.98	
Average Power	0.56	0.20	0.38			
BDEM – ‘Very’ Toxic						
FlatBiom	1.00	0.99	0.99	253.76	395.50	383.75
VeryLowBiom	0.00	0.00	0.00	584.35	1047.75	1024.75
LowBiom	0.85	0.00	0.83	459.10	948.25	905.00
ModerateBiom	0.91	0.00	0.90	292.89	668.17	541.54
HighBiom	1.00	0.99	0.99	252.51	484.00	312.25
Null	0.05	0.01	0.01	745.00	1065.25	1065.25
Average Power	0.75	0.40	0.74			
BDEM_T – ‘Very’ Toxic and One Test						
FlatBiom	0.99	0.99	0.68	370.74	395.50	383.75
VeryLowBiom	0.00	0.00	0.00	810.61	1047.75	1024.75
LowBiom	0.83	0.72	0.63	817.79	948.25	905.00
ModerateBiom	0.90	0.84	0.70	520.75	668.17	541.54
HighBiom	1.00	1.00	0.79	303.66	484.00	312.25
Null	0.06	0.01	0.01	650.00	1065.25	1065.25
Average Power	0.74	0.71	0.56			
BDEM_T – ‘Very’ Toxic and Double Lower Test						
FlatBiom	0.98	0.98	0.66	374.11	395.50	383.75
VeryLowBiom	0.00	0.00	0.00	1132.60	1047.75	1024.75
LowBiom	0.73	0.71	0.62	961.71	948.25	905.00
ModerateBiom	0.89	0.88	0.68	562.08	668.17	541.54
HighBiom	0.98	0.98	0.77	305.24	484.00	312.25
Null	0.00	0.00	0.00	NA	1065.25	1065.25
Average Power	0.72	0.71	0.55			
BDEM_T – ‘Very’ Toxic and Double Lower and Upper Test						
FlatBiom	0.98	0.98	0.66	374.11	395.50	383.75
VeryLowBiom	0.00	0.00	0.00	1125.00	1047.75	1024.75
LowBiom	0.73	0.71	0.62	956.23	948.25	905.00
ModerateBiom	0.71	0.71	0.67	512.57	668.17	541.54
HighBiom	0.76	0.76	0.75	253.95	484.00	312.25
Null	0.00	0.00	0.00	NA	1065.25	1065.25
Average Power	0.64	0.63	0.54			
BDEM_O – ‘Very’ Toxic						
FlatBiom	1.00	0.80	0.80	322.97	401.25	394.00
VeryLowBiom	0.00	0.00	0.00	814.19	1100.75	1081.00
LowBiom	0.93	0.03	0.83	700.97	1015.25	965.50
ModerateBiom	0.97	0.14	0.75	458.21	708.25	552.50
HighBiom	0.99	0.95	0.74	358.22	543.25	310.50
Null	0.02	0.00	0.00	1318.18	1122.00	1122.00
Average Power	0.78	0.38	0.62			

**Table A.3.** Geometric mean of correct selections across scenarios at the end of Phase II using the design described in Section 3.3 for different values of $\sigma^{2}$ and $\sigma_{\mu }^{2}$ .

Geometric mean	$\sigma^{2}$	$\sigma_{\mu }^{2}$
0	0.001	0.001
0	0.001	0.01
0	0.001	0.1
0	0.001	1
0	0.001	$1.00\times 10^{-4}$
0	0.001	$1.00\times 10^{-5}$
0	0.001	$1.00\times 10^{-6}$
0	0.001	2
0	0.01	0.001
0	0.01	0.01
0	0.01	0.1
0	0.01	1
0	0.01	$1.00\times 10^{-4}$
0	0.01	$1.00\times 10^{-5}$
0	0.01	$1.00\times 10^{-6}$
0	0.01	2
0.27	0.1	0.001
0.26	0.1	0.01
0.25	0.1	0.1
0.26	0.1	1
0.00	0.1	$1.00\times 10^{-4}$
0.00	0.1	$1.00\times 10^{-5}$
0.00	0.1	$1.00\times 10^{-6}$
0.25	0.1	2
0.28	1	0.001
0.19	1	0.01
0.20	1	0.1
0.16	1	1
0.26	1	$1.00\times 10^{-4}$
0.11	1	$1.00\times 10^{-5}$
0.07	1	$1.00\times 10^{-6}$
0.16	1	2
0.30	$1.00\times 10^{-4}$	0.001
0.00	$1.00\times 10^{-4}$	0.01
0.00	$1.00\times 10^{-4}$	0.1
0.00	$1.00\times 10^{-4}$	1
0.23	$1.00\times 10^{-4}$	$1.00\times 10^{-4}$
0.17	$1.00\times 10^{-4}$	$1.00\times 10^{-5}$
0.33	$1.00\times 10^{-4}$	$1.00\times 10^{-6}$
0.00	$1.00\times 10^{-4}$	2
**0.47**	** $1.00\times 10^{-5}$ **	**0.001**
0.45	$1.00\times 10^{-5}$	0.01
0.45	$1.00\times 10^{-5}$	0.1
0.45	$1.00\times 10^{-5}$	1
0.44	$1.00\times 10^{-5}$	$1.00\times 10^{-4}$
0.40	$1.00\times 10^{-5}$	$1.00\times 10^{-5}$
0.41	$1.00\times 10^{-5}$	$1.00\times 10^{-6}$
0.46	$1.00\times 10^{-5}$	2
0.36	$1.00\times 10^{-6}$	0.001
0.37	$1.00\times 10^{-6}$	0.01
0.37	$1.00\times 10^{-6}$	0.1
0.37	$1.00\times 10^{-6}$	1
0.35	$1.00\times 10^{-6}$	$1.00\times 10^{-4}$
0.33	$1.00\times 10^{-6}$	$1.00\times 10^{-5}$
0.32	$1.00\times 10^{-6}$	$1.00\times 10^{-6}$
0.36	$1.00\times 10^{-6}$	2
0.27	2	0.001
0.19	2	0.01
0.17	2	0.1
0.12	2	1
0.25	2	$1.00\times 10^{-4}$
0.12	2	$1.00\times 10^{-5}$
0.08	2	$1.00\times 10^{-6}$
0.19	2	2

The max value is highlighted in **bold**.
